# The Security *Versus* Freedom Dilemma. An Empirical Study of the Spanish Case

**DOI:** 10.3389/fsoc.2022.774485

**Published:** 2022-02-08

**Authors:** Gonzalo Herranz de Rafael, Juan S. Fernández-Prados

**Affiliations:** ^1^ Sociology Department, University of Malaga, Málaga, Spain; ^2^ Sociology Department, University of Almeria, CEMyRI, Almería, Spain

**Keywords:** security, freedom, victimization, ideology, surveillance

## Abstract

One of the classic debates in public opinion, now more prevalent due to the COVID-19 pandemic, has been the dilemma between freedom and security. Following a theoretical review, this article sets out to establish the sociodemographic profiles and those variables that can correlate and/or explain the inclination towards one or the other, that is, the dependent variable “freedom-security,” such as victimization or the assessment of surveillance. The analysis is based on the results of a survey prepared by the Center for Sociological Research (CIS, in Spanish) and administered to a sample of 5,920 Spaniards. The conclusions indicate that the majority inclination is for security, especially among older men, with elementary education attainment level and right-wing ideology. Furthermore, although victimization correlated with the dependent variable, the perception of being a possible victim led to a preference for safety rather than the actual experience of having been a victim. Finally, the positive assessment of surveillance through technologies such as video cameras explains or is strongly associated with security, making it a promising line of research for future work and a means to improve the understanding of the analyzed dilemma.

## Introduction

The COVID-19 pandemic is not the first event that has forced public opinion to consider the dilemma of freedom versus security in a world dominated by the influence of so-called new information and communication technologies. Currently, technological control is provoking debates around the right to privacy in the context of the surveillance society (Lyon, 2018; Lyon and Wood, 2021).

There are precedents to the influence of information and communication technologies, the extent to which they can control or influence citizens and countries, and their effect on these actors when valuing one side over the other when balancing freedom versus security. By way of example, the following cases affected both the personal safety of private citizens and nation-states: the case of “Wikileaks” in 2006; the “Snowden” case in 2013; “Cambridge Analytica” case in 2014, the spying of Jeff Bezos by Saudi Arabia in 2019, or the most recent “Pegasus case” which was made public in 2021.

Currently, the incidence of the pandemic has had a more significant impact on control over citizens and a corresponding lower degree of freedom. An example of this is the research carried out by the Canadian Citizen Lab into internet censorship, wherein it analyzed how the Chinese authorities, through WeChat, used an artificial intelligence system capable of detecting the semantic meaning of texts. From 1 January to 15 February 2020, up to 516 keyword combinations were set to trigger censorship, automatically locking the server, and preventing further communication (Ruan et al., 2020). According to “The COVID-19 Civic Freedom Tracker” database, developed by “The International Center for Not-for-Profit Law” (www.icnl.org), most of the restrictions applied by States as a result of the pandemic are: an increase in powers related to surveillance of citizens; suspension of rights; control over information and delay of political elections. The Spanish case is even more severe regarding freedom of information and the press, given that the Spanish government commissioned a government body, the Sociological Research Center, to include in its February Barometer the possibility of limiting all information on the pandemic in official sources (González-Requena, 2020).

Given these antecedents, this research aims to analyze the dilemma based on the opinions, attitudes, and behaviors of Spaniards with regards to freedom and security. This is a continued and constant dilemma in the field of sociology and social sciences, starting from the analyzes on the change from materialist to postmaterialist values worldwide as stated by Inglehart (Inglehart, 1990; Inglehart, 2018a), and particularized for the case of Spain by Díez Nicolás (2011), Díez Nicolás (2020). The working hypothesis established by Inglehart (1977), widely verified in countless investigations, was that those societies and individuals when reaching higher levels of personal security, including a lower level of crime, and a higher level of economic security, tend to be oriented toward more libertarian or self-expressive values.

This trend, however, is not valid for all the countries, as shown by the different waves of the World Values Survey. In the case of Spain, there has been a decrease in the post-materialism index compared to the waves of 1990 and 2005, further verified by the most recent wave of 2014 (Díez Nicolás, 2020). This empirical inclination can be ascribed to factors that have changed the perception of citizens towards feeling greater personal insecurity, such as, among others, the irruption of jihadist terrorism, the increase in organized crime and crime in general, the greater flexibility of the labor market and job insecurity, the uncertainty about the future pension model, the increase in crime and insecurity related to the internet and social networks, the real estate market, or lately, the current global viral pandemic.

For this reason, we believe that, in the Spanish case, depending on their perception of security, citizens will choose a greater or lesser degree of freedom. In this regard, we believe that security takes precedence over a greater or lesser degree of freedom. In other words, security is, to a greater extent, is the dominant value over freedom. More specifically, and as a working hypothesis, we believe that historical, economic, geographical, or sociological influences and the perception that the majority of Spaniards have towards citizen insecurity determine that security be valued more highly than freedom. In this instance, citizen insecurity refers to crime and other types of insecurity such as economic, employment, health, or informational.

This study traces the most significant theories about security versus freedom. It presents an empirical investigation for the Spanish case, based on the 2016 CIS General Social Survey, where a descriptive analysis will be carried out based on the more significant sociodemographic and socioeconomic variables. Secondly, multiple regression models will carry out an explanatory analysis to discover if Spaniards prefer security over freedom using crime and victimology as a dependent variable in terms of perception, opinion, attitude, and experience.

## Freedom *Versus* Security

In a globalized world, the interrelation and connectivity of countries, economies, and citizens are constant. In this order of things, it is observed that the private sphere is ever decreasing, resulting in a smaller margin of freedom, either for individuals or collectives, whereby citizens, in general, cannot control their information themselves, and the privacy of their information is constantly threatened. There is a general perception about the vertiginous social changes, hence the data mentioned above from the World Values Survey on the orientation of the most developed countries in recent waves towards more materialistic, scarcity, or survival values instead of values related to postmaterialism, self-expression, or emancipation (Inglehart, 2018b).

Our research does not focus on the classic six or twelve items of materialist/postmaterialist values but rather on the debate between, on the one hand, freedom and accessibility to surveillance information and, on the other hand, security related to surveillance linked to citizen security, such as personal security against crime and victimology. The research question for the Spanish case is: Do Spaniards, in general, perceive a greater degree of citizen insecurity and thus accept lower degrees of freedom in return? or simply stated; Do Spaniards demand higher levels of security measures because they feel insecure?

It is not easy to define the concept of freedom in philosophical terms, as it is a polyhedral and contradictory word. However, the type of freedom at stake is easier to define since it affects the collective. Two examples of freedom from the territorial and evaluative perspectives are the differing visions of American and European liberalism (Leonard, 2011) or Bauman’s consumerist interpretation of capitalist liberalism (Bauman, 1989). Similarly, differences of perspective could be included from the academic stance of authors such as Bay (1958), Sen (2001), Skinner (2012), Honneth (2015).

The term freedom is contradictory and difficult to apply to specific realities and is even more complicated when combined with the term security. In this sense, the questions posed are: what freedom? Freedom for whom? How much freedom? Freedom for what purpose? Inversely, the questions posed could be: what security? Security for whom? or how much security? Or even, security for what purpose?

Suppose we place ourselves in the classic dilemma, positive versus negative freedom (Berlin, 2002; Rothbard, 2015) or, more recently, quantitative versus qualitative freedom (Dierksmeier, 2019). In that case, it is observed that the object of freedom passes from the individual/property dyad to an individual triad/own good/other people’s good.

In this instance, we understand freedom as being able to carry out any individual or collective initiative, without any limitations or coercion, whether by the State or other individuals, and with budgets and objectives that reinforce both one’s own good and that of others. With this definition in mind, we believe that we can answer the questions previously formulated.

The concept of security has a similar or even greater number of facets as that of freedom. The most classic issue is that there are different types of security, national or state security, which ensures the protection of State, and human security, which ensures the protection of individuals (Mack, 2005; Krause and Williams, 2016). Logically, to the two types of security mentioned above, the supranational system that is increasingly important in the globalized world should also be added. Similarly, these supranational entities, together with nation-states, would also become subjects responsible for security. To these entities, we could also add other new actors such as NGOs or public opinion.

References to national or state security or supranational security are logically interrelated. The denomination of collective security seems more logical. In December 2004, the High-level Panel of the United Nations Secretary-General on Threats, Challenges, and Change presented a report entitled: “A more secure world: our shared responsibility.” The report highlights six groups of threats to collective security: conflicts between states; violence within the state (civil wars, human rights abuses, and genocide); poverty; infectious diseases; environmental degradation; nuclear, radiological, chemical, and biological weapons; terrorism; and transnational organized crime (Morillas, 2007). The UN Secretary-General, K. Annan, also pointed to the March 2005 document entitled: “In larger freedom: Towards Development, Security and Human Rights for All,” highlighting in point IV, “Freedom from Fear,” that most of the victims of these new conflicts are civilians.

The above notwithstanding, the discussion remains constant, whether in reference to state security or human security. With regards to the former, many believe that the State is predominant in matters related to security as it is the institution which must ensure it. Although individual citizens remain a definitive reference in this matter, it is the State that provides the necessary framework for the security of all. In the latter case, although human security is essentially focused on protecting individuals, there are two variants: the focus on “freedom from wants” and the “freedom from fear.” In the first, human security is based on basic human needs, or more specifically, on threats to well-being in the spheres of human rights, religion, poverty, hunger, disease, epidemics, the environment, wars, education, and information. In the second, human security revolves around the elimination of all types of coercion, threat, and violence in the daily lives of individuals (Suhrke, 1999; Seiple and Hoover, 2004; Knox Thames et al., 2009; Seiple et al., 2015).

Bauman’s sociological theory of liquid modernity and the nature of community extends the debate. Individuality increases freedom but does so at the expense of security and a sense of community. The concepts of “freedom versus security” and “individuality versus community” are simultaneously complementary and contradictory. Increasing either freedom or security comes at the expense of the other. The conflict between “security and freedom” and between “community and individuality” may never be resolved, but as they are equally indispensable values, we continue to search for a solution (Bauman, 2000, Bauman, 2001). In this sense, achieving a balance between freedom and security is probably impossible. The problem, however, is that when security is lacking, free agents are deprived of the trust without which freedom can hardly be exercised. When, on the contrary, it is freedom that is lacking, security feels like slavery or a prison (Bauman, 2005).

In methodological terms, in this research, we will consider information through the new information and communication technologies, which would essentially fit into the field of human security, both with regards to “freedom from want” and “freedom from fear.” Therefore, we understand security to be the central value that encompasses both the structure of human needs and its limitations due to coercion and threats in the daily lives of individuals.

These definitions align with United Nations Development Programme (1994) and more specifically with the idea that freedom also includes security. However, in operational terms, we believe that, among others, fear, insecurity, coercion of religious freedom, hunger, crime, epidemics can constrain citizens, essentially because the survival instinct is more fundamental than freedom. As Kofi Annan, Secretary-General of the United Nations, states in his report entitled: “In larger freedom: Towards Development, Security and Human Rights for All” of 21 March 2005, in point 14 of the document: “The notion of larger freedom also encapsulates the idea that development, security, and human rights go hand in hand” (Annan, 2005). Grim and Finke (2012), in an empirical investigation in 200 countries, observed that when governments and various social groups restrict religious freedom, the possibilities of violent persecution, conflicts, instability, and terrorism increase.

## Materials and Methods

This work is based on the descriptive analysis of a survey administered in Spain at the start of 2016 in which the behavior of the dependent variable “freedom or security” is analyzed.

The survey research was carried out by the Sociological Research Center (CIS) on a representative sample of adult Spaniards (see Table 1). The sample selection is based on a vast network of sampling points by municipality and a multi-staged sample selection system, culminating in face-to-face interviews. The sampling error was ±1.4% for the whole of the corresponding sample. All the methodological information of the survey, such as the technical sheet, questionnaire, data matrix, and descriptive results, are available for download in the corresponding link (see Table 1).

**TABLE 1:** Factsheet of the survey.

Study	CIS N^º^ 3123 Spanish general social survey
Scope	National (excluding Ceuta and Melilla)
Universe	Spanish population, both sexes, 18 years and over
Sample	5,290 interviews
Allocation	Non-proportional
Sampling points	523 municipalities and 48 provinces
Sampling procedure	Two stages and stratified by cluster. The selection of primary sampling units (sections) is proportional to the resident population, and the selection of final units (individuals) is carried out through a systematic selection of individuals residing in the area by house number
Sampling error	For a confidence level of 95.5% (two sigma) and where P = Q, the real error is ±1.4% for the whole sample and for the simple random sampling association
Date	From 22 December 2015 to 12 April 2016
URL	http://www.cis.es/cis/opencm/ES/1_encuestas/estudios/ver.jsp?estudio=14252

Source: The authors based on CIS, study 3,123 (2016).

The instrument or questionnaire presents the study variable (“freedom-security”) in the following literal way: “On a scale of 0–10, in which 0 means having full access to information even if it meant losing security, and 10 means having maximum security even if it meant losing access to information. Where would you position yourself? [0 = Maximum access to information even if it meant losing security (Freedom); 10 = Maximum security even if it meant losing access to information (Security)].” The question or dependent variable used includes an attitude, a certain predisposition, or a simple opinion rather than values. The latter, according to Rokeach (1973) are important life goals or standards which serve as guiding principles in a person’s life, while attitudes are learned predisposition to respond in a consistently favorable or unfavorable manner with respect to a given object (Fishbein and Ajzen, 1975).

The independent variables used include, on the one hand, traditional classificatory sociodemographic variables such as gender, age, marital status, subjective social class, ideology, education, size of locality, income, national identity, and religion. On the other, a set of questions related to security such as victimization, having been a victim of a crime, reporting a crime, having engaged in delinquent behavior in youth, and the perception of potentially being a victim of a crime (Herranz and Fernández-Prados, 2019); and other questions related to freedom of information or privacy such as internet use and assessing the presence of video cameras in public spaces.

The data analysis includes a descriptive, correlational, and explanatory methodology of the dependent variable being studied and is presented in three sections of the results. The descriptive analysis aims to draw a profile according to the sociodemographic variables and other “freedom-security” dilemma issues. The correlational analysis shows the relationships between those variables of a continuous nature with the study variables and their orientation (either towards greater security or towards greater freedom). Finally, a table with two multiple regression models is presented in the explanatory analysis, one with all the outstanding independent variables and the other with only those deemed to be significant.

## Results

### Descriptive and Profile

Table 2 presents the relationship between sociodemographic and socioeconomic variables and their frequency or percentage according to the sample of their values or alternative responses in some grouped cases. Thus, the survey sample is composed mainly of women (51.5%) who are over 60 years old (28.4%), married (55.6%), middle subjective social class (70.4%) with centrist ideology (33.9%), describing the most representative social characteristics of the Spanish population.

**TABLE 2:** Descriptive and sociodemographic profiles.

	Frec (%)	Freedom-security		
		*M* (0–10)	*SD*	Trend
Sex				
Male	48.5	6.6	2.28	Security
Female	51.5	6.2	2.42	Freedom
Age				
From 18 to 30	16.6	5.8	2.35	Freedom
From 30 to 45	27.3	6.3	2.35	
From 46 to 60	27.6	6.5	2.32	
Over 60	28.4	6.9	2.29	Security
Marital status				
Single	31.2	5.9	2.41	Freedom
Married	55.6	6.6	2.27	
Other (divorced)	13.1	6.7	2.44	Security
Social class				
Low	23.5	6.5	2.38	Security
Middle	70.4	6.4	2.35	
High	3.4	6.3	2.46	Freedom
Ideology				
Left	27.9	5.9	2.51	Freedom
Centre	33.9	6.5	2.24	
Right	12.4	6.8	2.3	Security
Education				
Primary	22	7.1	2.22	Security
Secondary	52.7	6.4	2.32	
Higher education	24.7	6.0	2.44	Freedom
Habitat				
Less than 10.000	21.8	6.5	2.34	Security
From 10.000 to 100.000	40.3	6.4	2.34	
Over 100.000	37.9	6.3	2.39	Freedom
Income				
Low (<1.200)	27.9	6.7	2.34	Security
Middle (1.200–2.400)	25.4	6.4	2.38	
High (>2.400)	15.3	6.0	2.39	Freedom
National Identity				
More Spanish	21.2	6.4	2.35	
As Spanish as nationalist	53.3	6.5	2.3	Security
More nationalistic	16.5	6.2	2.46	Freedom
Religion				
Non-believer (agnostic. atheist)	21.9	5.6	2.55	Freedom
Non-practicing believer	45.4	6.6	2.28	
Practicing believer	31.9	6.8	2.19	Security
Total	100.0	6.4	2.36	

Source: The authors based on CIS, study 3,123 (2016).

Table 2 also contains the descriptive analysis, mean and standard deviation, of the dependent variable “freedom-security” for each of the sociodemographic and socioeconomic characteristic values of the sample. For the sample as a whole, the mean of the “freedom-security” variable is 6.4 on a scale of 0–10 with a standard deviation of 2.36. In essence, this means that Spanish population leans towards “security.” The profile where security tends to stand out corresponds to that of men (M = 6.6; SD = 2.28); over 60 years old (M = 6.9; SD = 2.29); widowed, divorced or separated (M = 6.7; SD = 2.44); low subjective social class (M = 6.5; SD = 2.38); with right-wing ideology (M = 6.8; SD = 2.3); elementary education (M = 7.0; SD = 2.22); rural locality (M = 6.5; SD = 2.34); low income (M = 6.7; SD = 2.34); identified as a Spanish national (M = 6.5; SD = 2.30) and practicing Catholic (M = 6.8; SD = 2.19).

Table 3 also shows the description of the variables related to safety or victimization and freedom or privacy that appear in the questionnaire. Thus, half the respondents said they had been the victim of a crime (50.7%), a third had reported a crime (33.6%), two-fifths had engaged in delinquent or quasi-criminal behavior in adolescence (22%), and a 10th considered they were likely to be the victim of a crime (9.4%). Likewise, the vast majority considered surveillance cameras in public spaces to be very good (37.7%) or good (46.6%), and finally, almost three-quarters used the internet (72.3%), and almost half the respondents used social networks (48.2%).

**TABLE 3:** Descriptive and sociodemographic profiles.

	Frec (%)	Freedom-security		
		*M* (0–10)	*SD*	Trend
Victim				
Never	49.2	6.6	2.29	Security
1 time	30.4	6.3	2.38	
2 or more times	20.3	6.1	2.45	Freedom
Complaint				
Never	66.4	6.5	2.34	Security
1 time	23.1	6.3	2.33	
2 or more	10.5	6.1	2.49	Freedom
Deviation				
Never	78.0	6.6	2.29	Security
1 time	11.0	6.1	2.41	
2 or more	11.0	5.7	2.58	Freedom
Victimizable				
Yes	9.4	6.7	2.38	Security
No	90.6	6.3	2.36	Freedom
Video cameras				
Very good	37.7	6.8	2.26	Security
Good	46.6	6.3	2.29	
Bad or very bad	9.2	5.4	2.59	Freedom
Internet				
Do not use	28.4	7.1	2.17	Security
Uses internet (mail only)	23.1	6.5	2.42	
Use internet (social networks)	48.2	6.1	2.35	Freedom
Total	100.0	6.4	2.36	

Source: The author based on CIS, study 3,123 (2016).

As in the previous table, the means and standard deviations for each variable are presented with the values of the independent variables that lean towards either security or freedom are highlighted. Thus, the profile of those surveyed with higher means and, therefore, lean more towards security are those who had never been victim of a crime (M = 6.6; SD = 2.29); never reported a crime (M = 6.5; SD = 2.34); nor engaged in pre-delinquent behaviors in adolescence (M = 6.5; SD = 2.29); although they did consider that they were likely to be a victim of a crime (M = 6.7; SD = 2.36); strongly agreed with surveillance cameras (M = 6.8; SD = 2.26) and did not use the internet (M = 7.1; SD = 2.17).

### Correlation Among Continuous Variables

The results of the correlation matrix between the dependent variable, “freedom-security,” and the continuous sociodemographic variables and those related to victimization and privacy are shown in Table 4. Only the variable “nationalism” does not correlate with the study variable, and all the others reach a significance of *p* value < 0.001 except for size of locality and religious practice with a *p* value < 0.01. Although it should be borne in mind that the n of the sample is high and can cause this significant correlation with most of the variables, we can point to certain co-variations between the dependent variable and the remainder. That is, a desire for greater levels of security is related to older age, lower social class, more right-wing ideology, lower educational attainment and living in smaller localities, and greater religious observance. In addition, the demand for greater security shows a lower correlation with having been the victim of a crime, reporting a crime, and having pre-criminal or delinquent behaviors, or manifesting stronger agreement with surveillance cameras and lower use of social networks.

**TABLE 4:** Correlation between Freedom-Security and continuous variables.

	PCC	Sig. (Bilateral)	*n*	To more security
Age	.168***	0.000	4,692	More age
Social class	−.045**	0.002	4,618	Less social class
Ideology	.156***	0.000	3,622	More right
Education	−.146***	0.000	4,483	Less education
Habitat	−.045**	0.002	4,692	Small habitat
Income	−.099***	0.000	3,208	Less income
National Identity	−.029	0.057	4,264	Not significant
Religious practice	.052**	0.002	3,584	More practical
Victim	−.095***	0.000	4,692	Less victim
Complaint	−.056***	0.000	4,692	Less reporting
Deviation	−.125***	0.000	4,692	Less deviation
Video cameras	−.187***	0.000	4,422	More agreement
Social media	−.168***	0.000	4,692	Less use

Source: The author based on CIS, study 3,123 (2016). Pearson correlation coefficient (PCC); **p values < .01; ***p values < .001.

### Explanatory and Regression Analysis

Finally, Table 5 shows the results of two multiple regression models where, on the one hand, all the variables used in the descriptive variables and the correlation are contemplated (with the insignificant variable of nationalism); and, on the other hand, only those variables that in the last step had proven to be significant in this multivariate technique. Thus, the first model is comprised of 15 variables attaining a low R squared (R^2^ = 0.068), and only five variables are significant within the model. The second model presents only those five significant variables in the final step. These reinforce the level of significance; they all reach *p* value < 0.001 and increase the R squared (R^2^ = 0.080). These variables confirm a first approximation to a more detailed explanatory profile or predictive variables that lean towards security, male gender, older age, right-wing ideology, low educational level, and supporting surveillance cameras in public spaces.

**TABLE 5:** Regression analysis multiple (Dependent variable = Freedom-Security).

	Model I				Model II		
	B	t	Sig		B	t	Sig
(Constant)	7.746	13.591	0.000	(Constant)	6.715	30.466	0.000
Sex	−0.508	−4.466	0.000	Sex	−0.440	−5.533	0.000
Age	0.014	3.184	0.001	Age	0.015	5.745	0.000
Social class	−0.017	−0.357	0.721	Ideology	0.113	6.026	0.000
Ideology	0.075	2.746	0.006	Education	−0.073	−5.604	0.000
Education	−0.052	−2.356	0.019	Video cameras	−0.440	−7.835	0.000
Habitat	−0.028	−0.777	0.437	—	—	—	—
Nationalism	−0.005	−0.093	0.926	—	—	—	—
Income	−0.010	−0.275	0.783	—	—	—	—
Religious practice	−0.027	−0.572	0.567	—	—	—	—
Victim	−0.044	−0.614	0.539	—	—	—	—
Complaint	0.038	0.428	0.669	—	—	—	—
Deviation	−0.004	−0.065	0.948	—	—	—	—
Victimizable	−0.193	−0.990	0.322	—	—	—	—
Video cameras	−0.433	−5.159	0.000	—	—	—	—
Social media	−0.048	−0.774	0.439	—	—	—	—
*R*	0.262	—	—	*R*	0.284	—	—
*R* ^2^	0.068	—	—	*R* ^2^	0.080	—	—

Source: The author based on CIS, study 3,123 (2016).

## Discussion and Conclusion

The principal hypothesis of the present study was that the majority trend of the population would lean towards security rather than freedom. This has been confirmed by the results in the case of Spain. In the seventh and last wave of the World Values Survey (2017–2021), which is still being developed, similar results are found for the set of 54 countries for which data was available, where 69.7% of the more than eighty thousand interviewees answered that security is more important than freedom. Only in three countries does freedom have a majority percentage: the United States, New Zealand, and Australia (Haerpfer et al., 2020). In this sense, comparisons with other international studies that include similar questions related to the freedom vs security debate such as the European Social Survey, International Social Survey Program (Fernández-Prados et al., 2019), as well as sociodemographic profiles and other social characteristics or explanatory factors could be helpful to confirm or expand this hypothesis and trend.

The study conducted is not able to give a definitive answer about future trends in the population’s preferences between freedom and security. Among other reasons, the research is Cross-sectional and not longitudinal, moreover, it is limited to an only country to the influence of a global context. Certainly, it would be necessary to conduct or analyse cross-sectional and international studies. The recent analysis of the World Values Survey shows a return to the values of loyalty, security primacy, distrust, and authoritarian populisms as a reaction to the values of tolerance and individual freedom (Norris and Inglehart, 2019). In short, culture seems to be facing the freedom-security dilemma as a historical pendulum, although our current context is priming security.

The relationship and correlation found in this article between victimization and the “freedom-security” debates provide at least two nuances. Firstly, against what is expected, people who are victims of crimes, whistle-blowers, and those who had delinquent or pre-criminal behaviours in adolescence lean more towards freedom than security, while those who are perceived as priority targets of crimes overwhelmingly opt for the security. That is, the issue of security has is related to personal experiences or behaviours, thus connecting them to the theory of securitization and de-securitization by Butler (2020), which states that the major security issues such as terrorism, climate change, gender violence, or any conflict are constructed and deconstructed in political discourses and public opinion. In this sense, the inclusion of more variables related to the perception of insecurity in future studies could also be helpful to build more significant explanatory models with a stronger association.

In addition to the sociodemographic variables in which the association with security rather than freedom have been verified (male gender, older age, and lower level of education attainment), ideology has behaved as a highly predictive variable, associating the right more towards security. In contrast, the left was associated more closely with freedom. Azmanova (2020) points to a redefinition of the ideological panorama and the left-right axis as a consequence of the impact of globalization in Western societies, with a winning party that considers it an opportunity and a losing party that perceives it as a risk. The winners and supporters of globalization value its advantages for a more cosmopolitan lifestyle and open economy, placing themselves in traditionally left-wing positions. The losers of globalization represent blue-collar workers, those who fear or are insecure about opening international markets and migration, defending positions of a certain economic patriotism, materialistic values, and ideological positions located to the right and extreme right (Azmanova, 2020).

Perhaps another fitting interpretation of the trend towards security comes from the interpretation of the consumer society, and by extension the network society, in the context of Bauman’s sociological theory. In liquid modernity, consumer society replaces groups with an increasing number of “swarms” and the comfort of flying in a swarm derives from having security in numbers. The individual is based on the idea that when many have chosen to fly in the same direction, it must be a good and safe choice. In a “swarm” there is no exchange, cooperation or complementarity; there is only physical proximity and basic coordination in a given direction. Swarms have no leaders and no hierarchy of authority. They gather, disperse and reassemble from one event to the next, drawn by shifting and moving targets. The actual leadership of the swarm may “assign” leadership roles to particular members for a short period of time before they return to anonymity within the “swarm” (Bauman, 2007).

The role of new technologies requires a reflection that Manuel Castells (1996) pointed out in the last century when he differentiated between the mere information society and the informational society. In other words, information and communication technologies have been the basis for entering a new informational era after the industrial society. This radical social and cultural change situates the debate on the dilemma between freedom and security precisely in the development and trends of technologies. Thus, the great historical and current challenge, according to Clarise Véliz (2020), is to recover individual and collective privacy (freedom) in the face of the data economy (security) in the hands of the power of large technology companies and governments. The fact of shifting the debate from a mere technological issue to the realm of power relations makes the freedom-security dilemma an exciting ideological and philosophical topic.

The theses and the consequences of the surveillance society proposed by David Lyon have been reflected in the solid and significant association between the assessment of the presence of cameras in public spaces and the “freedom-security” debate. The results have confirmed that the preference for security is supported by those who defend the presence of public social control tools such as surveillance cameras. The current crisis caused by the COVID-19 pandemic has sparked the debate on “freedom-security” and the new mechanisms of social control such as mobile phones and tracking and surveillance applications used by States and technology companies (Taylor et al., 2020). In this way, the virtual space acquires an increasing relevance to address the redefined dilemma as privacy-cybersecurity.

Finally, the current crisis caused by the global pandemic points to an emphasis on security and new social challenges to be faced at global, national, and individual levels (Varin, 2022). At the global level, it has increased tensions between the superpowers of China and the United States and demonstrated the unwillingness of rich countries to help much poorer countries when the health of their own populations is at risk. For countries, in some cases it has increased their tendency to fragment, and in others it has led to authoritarian rule that may well outlast the pandemic. And at the individual level, it has led to unprecedented forms of intervention, accentuating the growth of the “surveillance state” and “quarantining” rights and freedoms. In this context in which the pandemic and the measures adopted have led to greater confrontation, polarization and socio-political control, the debate between security and freedom takes on greater interest and connotations of a political and ideological nature from the point of view of public perception and opinion (Fernández-Prados et al., 2021). In this way, the new context of the global pandemic crisis, the dilemma between freedom and security, and public opinion become a triad that will undoubtedly generate future lines of research.

## Data Availability

The original contributions presented in the study are included in the article/Supplementary Material, further inquiries can be directed to the corresponding author.
